# Deoxynivalenol (DON) Contamination of Feed and Grinding Fineness: Are There Interactive Implications on Stomach Integrity and Health of Piglets?

**DOI:** 10.3390/toxins9010016

**Published:** 2017-01-01

**Authors:** Sven Dänicke, Andreas Beineke, Andreas Berk, Susanne Kersten

**Affiliations:** 1 Institute of Animal Nutrition, Friedrich-Loeffler-Institute (FLI), Federal Research Institute for Animal Health, Bundesallee 50, D-38116 Braunschweig, Germany; andreas.berk@fli.de (A.B.); susanne.kersten@fli.de (S.K.); 2 Institute of Pathology, University of Veterinary Medicine Hannover, Foundation, Bünteweg 17, D30559 Hannover, Germany; andreas.beineke@tiho-hannover.de

**Keywords:** deoxynivalenol, pig, feed grinding fineness, stomach pathology

## Abstract

The common feed contaminant deoxynivalenol (DON) was reported to influence the morphology of the *pars nonglandularis* (PN) of porcine stomach. Moreover, finely ground feed is known to trigger the development of ulcers and other pathologies of PN while coarsely ground feed protects from such lesions. The interactions between grinding fineness and DON contamination of feed were not examined so far. Therefore, both finely and coarsely ground feeds were tested either in the absence or presence of a DON contaminated wheat on growth performance and health of rearing piglets, including stomach integrity. DON contamination significantly reduced feed intake and serum albumin concentration with this effect being more pronounced after feeding the coarsely ground feed. Albeit at a higher level, albumin concentration was also reduced after feeding the finely ground and uncontaminated feed. Finely ground and DON-contaminated feed caused a significantly more pronounced lymphoplasmacytic infiltration both of PN and *pars glandularis*, partly paralleled by lymph follicle formation and detritus filled *foveolae* and tubes suggesting a local immune response probably triggered by epithelial lesions. It is concluded that DON contamination of feed exacerbates the adverse effects of finely ground feed on stomach mucosal integrity.

## 1. Introduction

Deoxynivalenol (DON) is a common contaminant of pig feed mainly originating from *Fusarium*-infected cereal grains. Under practical feeding conditions, most prominent toxic effects of DON include a decrease in feed intake causing a reduced live weight gain and contributing to the DON-associated immune-modulation [1]. Besides, DON effects on stomach weight and pathology have been reported [2,3,4,5,6]. Gross macroscopical findings of the *pars nonglandularis* (*seu pars oesophagea*, *pars proventricularis*) were described as a less extended area, a thicker appearance and a smaller degree of folding [4,6]. These alterations were suggested to reduce the susceptibility of pigs to ulceration [6]. 

Porcine stomach ulcers predominantly affecting the *pars nonglandularis* are a common organ pathology observed in modern pig production systems. Evaluations from slaughterhouses suggest that 20% of pigs show erosions and further 60% pre-ulcerative parakeratotic lesions [7]. Amongst others, feed particle size was described as an important predisposing factor for gastric ulcera [7]. Feed particle size influences mixing of gastric chyme and stomach filling whereby finely ground feed accelerates stomach emptying and increases the time in which *pars nonglandularis* remains unprotected from hydrochloric acid and fundus and duodenum originating digestive juices [7]. Not only an accelerated gastric emptying due to feeding finely ground feed but also the gastric filling itself has been identified as main risk factors for the development of gastric lesions [7]. As DON contamination of feed decreases feed intake, it might be assumed that gastric filling is also reduced. Taking into consideration the anorectic effect of DON and the mentioned DON-associated gross-macroscopical alterations of the *pars nonglandularis* it seems reasonable to hypothesize that DON might interfere with the feed particle size in developing stomach lesions in pigs. Besides possible implications of grinding fineness and DON contamination on stomach integrity, other nutritional consequences also have to be considered. For example, due to coarse ground feed a higher proportion of undigested nutrients might be shifted to the hindgut [8] giving rise to an altered fermentation pattern in this segment. Moreover, *Fusarium* infection associated physico-chemical alterations, including cell-wall pre-digestion and altered enzymatic activities of grain, could also contribute to regional changes in nutrient digestion and fermentation [9,10]. Therefore, main endproducts of microbial fermentation such as volatile fatty acids (VFA) in chyme of small and large intestine might reflect alterations in the sites of nutrient utilization. 

To test these hypotheses, two diet types differing in particle size distribution, either in the absence or presence of a *Fusarium* toxin contaminated wheat containing mainly DON, were fed to rearing piglets. As endpoints we investigated growth performance, fermentative pattern in intestinal chyme and health parameters, including gross-macroscopic appearance and histological lesions of stomach.

## 2. Results

### 2.1. Particle Size Distribution of the Diets

Using two different hammer mill screen sizes for grinding the feedstuffs of the two diet types resulted in clearly different particle size distributions. Finely ground feed contained approximately twice as much particles lower than 125 µm in size compared to coarsely ground feed (Table 1) while larger particles (>710 µm) were approximately 1/3 less abundant in the finely ground diets. Inclusion of *Fusarium* toxin contaminated wheat instead of control wheat appeared to increase the proportion of large particles (>710 µm) at the expense of small particles (<125 µm). Interestingly, this effect occurred when feedstuffs were ground both with the small and large sized hammer mill screen.

### 2.2. Nutrient Composition and DON Concentration of the Diets

Irrespective of grinding fineness, small differences in nutrient composition were noticed between the diets containing control wheat and *Fusarium* toxin contaminated wheat. Therefore, neutral detergent fiber (aNDF_OM_) content of the diets containing the *Fusarium* toxin contaminated wheat was slightly lower compared to the diets containing the control wheat.

Preliminary DON analysis of the contaminated wheat resulted in a concentration of 20 mg/kg whereby a theoretical DON concentration of 5 mg/kg diet was expected. The analyzed DON concentrations were slightly lower than the targeted DON concentration (−1.4 and −1.6 mg/kg for FUS-fine and FUS-coarse, respectively).

### 2.3. Growth Performance

Feed intake was significantly stimulated in piglets fed the CON-coarse diet compared to both FUS-diets within the 1st 3 weeks of the experiment (Table 2). During the last two weeks of the study the piglets of group CON-fine consumed more feed than piglets fed the FUS-diets. Taking the whole five weeks together, both control groups consumed significantly more feed than their FUS-fed counterparts. Live weight gain mostly mirrored the differences in feed intake between the groups albeit the significance relationships slightly differed. Here, the feed structure tended to influence live weight gain in the last two weeks of the experiment (*p* = 0.062) in addition to the significant effect of the cereal type (*p* = 0.001). Feed to gain ratio was significantly influenced by feed structure and in tendency by cereal type in an interactive manner (*p*_CTxStr_ = 0.092) when evaluated over the whole experiment. Feeding of FUS-coarse diet significantly increased feed to gain ratio compared to FUS-fine while such an effect was not observed in the CON-diets.

### 2.4. Blood Clinical-Chemical Traits and DON Residues

While total bilirubin concentration, ASAT, GLDH and GGT activities remained uninfluenced, total protein and albumin concentration were significantly decreased in piglets fed the FUS-diets (Table 3). Moreover, feed structure additionally decreased albumin concentration in piglets fed the FUS-coarse diet when compared to the FUS-fine diet. A similar feed structure related effect was observed in the CON-coarse group compared to their CON-fine counterpart albeit this decrease occurred at a higher level. DON and de-DON concentrations in serum clearly reflected the cereal type without any further influence of the feed structure.

**Figure 1:**
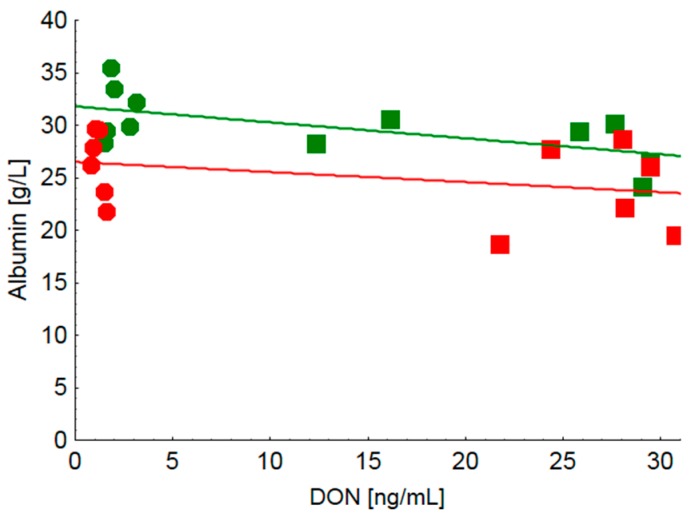
Relationship between blood concentrations of deoxynivalenol (DON) and albumin of weaned piglets fed a fine or coarse ground uncontaminated control diet (CON) or a *Fusarium* toxin contaminated diet (FUS). CON-fine ●, CON-coarse ●, FUS-fine ■, FUS-coarse ■; Albumin (finely ground diets, *N* = 12) = 31.8 − 0.152^a^·*x*; *r*^2^ = 0.38, Albumin (coarsely ground diets, *N* = 12) = 26.5 − 0.096^b^·*x*; *r*^2^ = 0.12, ^a,b^ slopes are significantly different (*p* < 0.05).

Because both grinding fineness and cereal type affected serum albumin concentration distinctively, the relationships between serum albumin concentration and serum DON concentration as an indicator of DON exposure resulting from the consumption of the FUS-diets was examined regressively. Here, two linear regression analyses were performed, including either both finely ground diets (CON-fine and FUS-fine) or both coarsely ground diets (CON-coarse and FUS-coarse). The results revealed that the albumin concentration decreased significantly more steeply with each increase in serum DON concentration for groups fed the finely ground diets compared to their coarsely ground counterparts (Figure 1).

### 2.5. Cell Culture Tests

**Figure 2:**
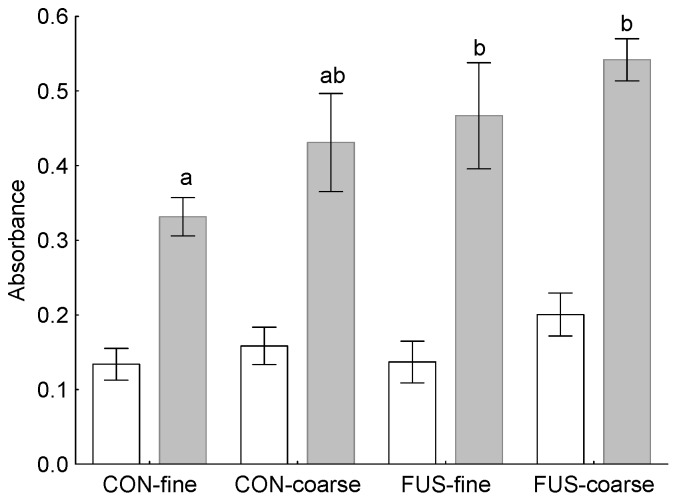
Absorbance of unstimulated (*p*_CT_ = 0.397, *p*_Str_ = 0.105, *p*_CTxStr_ = 0.460) and concanavalin A-stimulated (*p*_CT_ = 0.028, *p*_Str_ = 0.109, *p*_CTxStr_ = 0.817) peripheral blood mononuclear cells (PBMC) isolated from weaned piglets fed a fine or coarse ground uncontaminated control diet (CON) or a *Fusarium* toxin contaminated diet (FUS) (*n* = 6) in the MTT (3-[4,5-dimethylthiazol-2-yl]-2,5-diphenyl-tetrazolium bromide)-assay (570 nm); ^a,b^ different letters close to whiskers indicate significant differences (*p* < 0.05).

Absorbance of unstimulated peripheral blood mononuclear cells (PBMC) was not influenced by treatments while ConA-stimulation resulted in significantly higher absorbance due to feeding the FUS-diets (*p*_CT_ = 0.028) and a tendency for an increase in groups fed coarsely ground diets (*p*_Str_ = 0.109, Figure 2). The corresponding stimulation indices differed not significantly between groups and amounted to 2.9 ± 1.5, 2.8 ± 0.7, 4.3 ± 2.2 and 3.0 ± 1.1 in groups CON-fine, CON-coarse, FUS-fine and FUS-coarse, respectively.

### 2.6. Ingesta pH-Values

pH-values were neither influenced by cereal type nor by feed structure (Table 4). On average, pH-value in stomach chyme amounted to 3.55 and increased continuously from duodenum to jejunum and ileum to a value of 6.63. In caecum, a drop of approximately 0.76 pH-units compared to ileum was noticed while pH-values increased again in colonic chyme and rectal feces to 6.33 and 6.54, respectively.

### 2.7. Microbial Fermentation Profile in Jejunum and Caecum

Total volatile fatty acid (VFA) concentrations were approximately 6-fold higher in caecal chyme compared to jejunal ingesta when evaluated across treatments (Table 5). Inclusion of *Fusarium* toxin contaminated instead of uncontaminated control wheat in the diets resulted in an approximately 38% lower concentration of total VFA in caecal chyme while such effects were not observed in jejunum. This explained the significant interactions between intestinal segment and cereal type. Feed structure was without influence with regard to VFA. In contrast, the fermentative pattern was solely influenced by the intestinal segment with the exception of the proportion of butyric acid (C4). While in caecum the proportion of acetic acid (C2) of total VFA was approximately 23% lower compared to jejunum, the share of propionic acid (C3) was 133% higher in caecum. The butyric acid proportion was comparable in jejunum and caecum but was influenced by an interaction between intestinal segment and feed structure caused by an increase after feeding the coarsely ground control diet while the opposite was observed in the diets containing the *Fusarium* toxin contaminated wheat. Interestingly, this effect was detected both in jejunum and in caecum. The percentages of the minor fatty acids valeric acid (C5), iso-C5 and iso-C4 were approximately 11.4, 2.3 and 2.2-fold higher in caecum than in jejunum and remained uninfluenced by treatments.

### 2.8. Organ Weights

Of the relative organ weights, only the empty stomach weight was significantly influenced by treatments (Table 6). Feeding of the coarsely ground diets resulted in an increased weight whereby this effect appeared to be more pronounced when the diet FUS-coarse was fed. 

### 2.9. Gross Macroscopical Evaluation of Stomach Mucosa and Histopathology of Pars nonglandularis and Pars glandularis

Gross macroscopical inspection of stomach mucosa revealed no obvious treatment effects. Only 1 pig out of group CON-fine, 2 pigs of group CON-coarse and 1 pig of group FUS-coarse were characterized by mild changes, partial bile staining and hyperkeratosis in the *pars nonglandularis* (score 1) while all other pigs, including group FUS-fine, were visually inconspicuous. 

**Figure 3:**
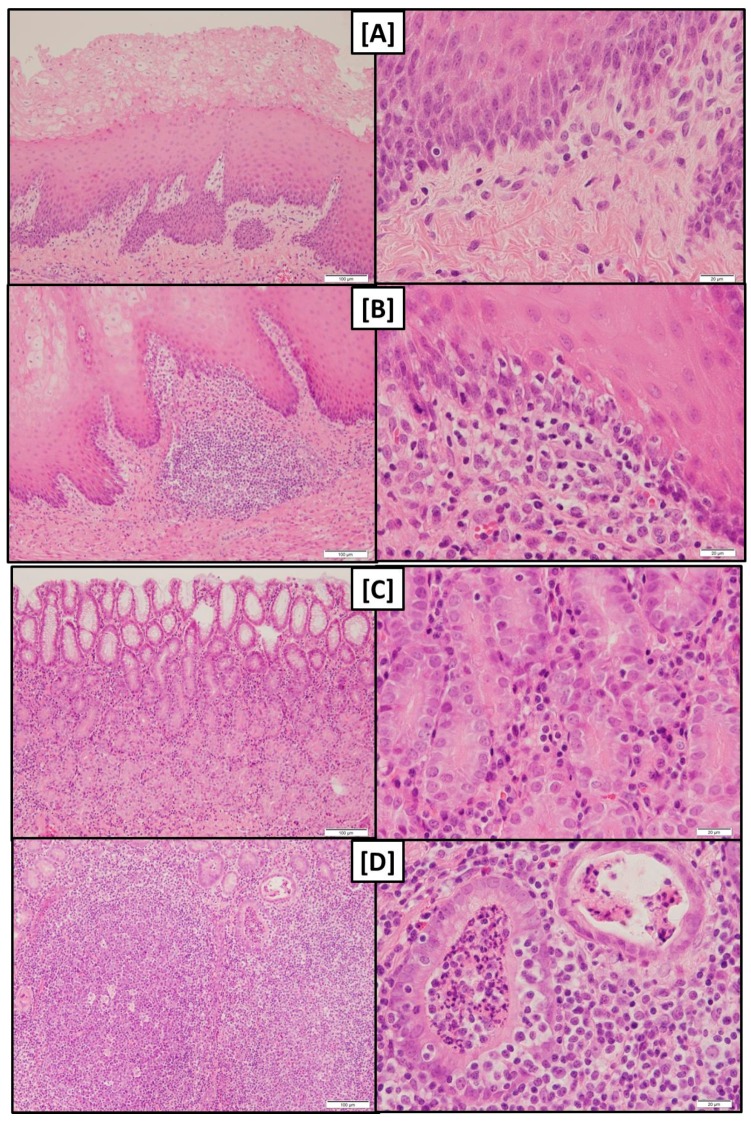

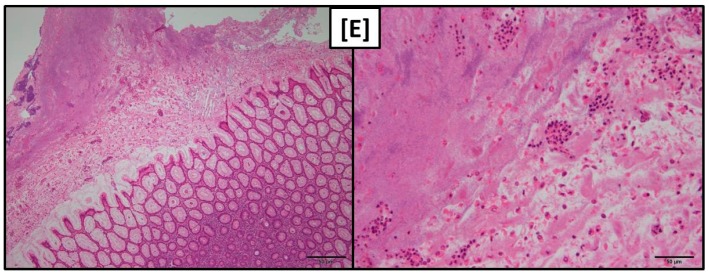
Examples of haematoxylin and eosin stained sections of stomach: (**A**) *pars nonglandularis*—low degree lymphoplasmacytic infiltration (normal finding); (**B**) *pars nonglandularis*—high degree lymphoplasmacytic infiltration; (**C**) *pars glandularis*—low degree lymphoplasmacytic infiltration (normal finding); (**D**) *pars glandularis*—high degree lymphoplasmacytic infiltration and detritus filled *foveolae* and tubes (group FUS-fine); and (**E**) intraluminal accumulation of fibrinous material with neutrophilic granulocytes, partially as aggregates, and bacteria (group FUS-fine). Magnification: (**A**–**D**) left ×10, right ×40; (**E**) left ×4, right ×20.

In contrast to gross macroscopical findings, *pars nonglandularis* and *pars glandularis* of pigs fed FUS-fine were significantly more infiltrated by lymphocytes compared to group FUS-coarse while a higher scoring variation was noticed within group CON-fine (Table 7). In both stomach regions, the lymphoplasmacytic infiltration resulted in follicular structures partially extending from *epithelium mucosae* to *tela submucosa* and *tunica muscularis* (Figure 3). Such follicles were more abundant in groups FUS-fine and CON-fine than in group FUS-coarse.

Two pigs of group FUS-fine and one of group CON-fine impressed by several detritus filled *foveolae* and tubes (Figure 3) in the *pars glandularis*. In addition, in one of the two affected pigs of group FUS-fine, an intraluminal accumulation of fibrinous material with neutrophilic granulocytes, partially as aggregates, and bacteria was noticed (Figure 3).

### 2.10. Principal Component Analysis (PCA)

The PCA was performed to visualize possible relationships between 15 variables previously shown to be significantly influenced by treatments or closely related to the significant parameters. PCA was based on correlations. The results revealed that the first two components (PC 1 and PC 2) extracted approximately 45% of the total variance. The scree plot as a visualization between successively extracted components and the corresponding eigenvalues showed a distinct break point after extraction of 5 principal components. This is accepted to indicate the transition from the most important components to those not contributing significantly to the total explained variance. Moreover, the eigenvalue of 1.0 as the mean value of all 15 eigenvalues also corresponded to a total of five extracted components that cumulatively explained approximately 76% of the total variance. 

**Figure 4:**
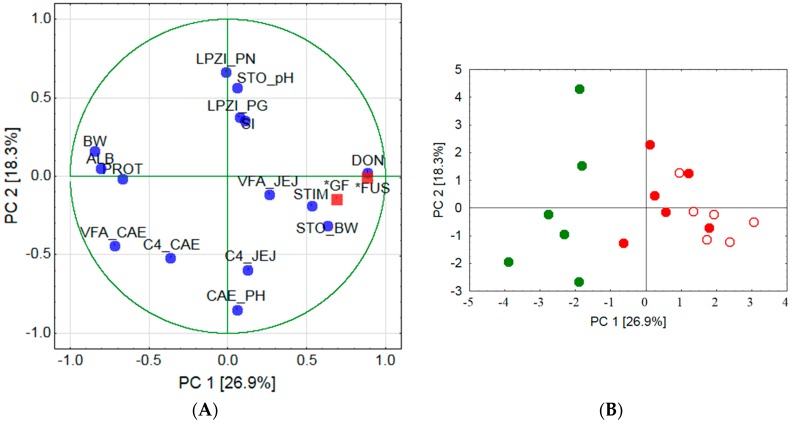
Principal Component (PC) Analysis for a two-dimensional visualization of the relationships between 15 variables collected from the experiment: Variables for the analysis: LPZI_PN, lymphoplasmacytic infiltration of *pars nonglandularis*; LPZI_PG, lymphoplasmacytic infiltration of *pars glandularis*; STIM, proliferation of peripheral blood mononuclear cells (PBMC) after Concanavalin A (ConA)-stimulation; SI, stimulation ability of PBMC after ConA-stimulation relative to unstimulated PBMC; C4_JEJ, butyric acid proportion in jejunal chyme; VFA_JEJ, volatile fatty acid concentration in jejunal chyme; C4_CAE, butyric acid proportion in caecal chyme; VFA_CAE, volatile fatty acid concentration in caecal chyme; PROT, serum protein concentration; ALB, serum albumin concentration; DON, serum deoxynivalenol concentration; BW, body weight at day 35 of experiment; STO, stomach weight relative to BW; STO_pH, pH-value in stomach chyme; CAE_PH pH-value in caecal chyme; Additional variables: *GF, grinding fineness; *FUS, *Fusarium* toxin contaminated diets, containing mainly DON. (**A**) Projection of variables; and (**B**) projection of cases (pigs): CON-fine ●, FUS-fine ●, FUS-coarse ○.

Projecting the additional variables into the variable space (Figure 4) showed that both *Fusarium* toxin contaminated diets, containing mainly DON, (*FUS) and grinding fineness (*GF) were highly positively correlated to PC 1 and clustered together with serum DON concentration (DON), proliferation of PBMC after ConA-stimulation (STIM) and stomach weight relative to body weight (STO). Further clusters were formed by lymphoplasmacytic infiltration of *pars nonglandularis* (LPZI_PN), lymphoplasmacytic infiltration of *pars glandularis* (LPZI_PG), stimulation ability of PBMC after ConA-stimulation relative to unstimulated PBMC (SI) and pH-value in stomach chyme (STO_pH) which correlated moderately positive with PC 2.

Body weight at day 35 of experiment (BW), serum protein concentration (PROT) and serum albumin concentration (ALB) formed a further cluster which correlated strongly negative with PC 1. Chyme characteristics correlated moderately negative with PC 2 and partly with PC 1.

Projecting the cases (i.e., individual pigs) based on the evaluated 15 variables clearly separated the CON-fine group from groups FUS-fine and FUS-coarse. The latter two groups apparently did not form distinct clusters (Figure 4). 

## 3. Discussion

DON is well known for its anorectic effects in pigs. Literature compilation revealed a decrease in feed intake by approximately 5% per each 1 mg increase in dietary DON concentration exceeding the critical dietary concentration of 0.9 mg/kg [1,11]. In the present experiment the piglet groups fed the DON-contaminated diets (FUS-fine and FUS-coarse) consumed approximately 20% less feed than their control counterparts over the entire experimental period of 35 days and roughly approached the predicted decrease. 

If a lower feed consumption is associated with a lower gut fill, then it seems reasonable to assume that general stomach filling would also be reduced in DON-fed piglets. Taking further into account that finely ground feed additionally contributes to an accelerated gastric emptying, group FUS-fine would be at a higher risk to develop gastric lesions than all other groups as gastric filling is generally considered as a risk factor [7]. This might be due to a disproportion between gastric acid production and a parallel depletion of the gastric buffering ingesta [12]. Stomach filling might be associated to the weight of this organ. In the present experiment, the observed higher relative stomach weights due to feeding coarsely ground compared to finely ground feed agree with literature reports (e.g., [13,14]). They are discussed as a reactive muscle hypertrophy resulting from the higher physical efforts to transport a chyme with a higher dry matter content as it has been observed after feeding coarsely ground feed [14,15]. The increased relative stomach weight due to feeding coarsely ground feed occurred at a higher level when the diet contained the DON-contaminated wheat. An increased relative stomach weight due to feeding of DON contaminated diets was also previously reported [2,6]. This effect might be due to the fact that piglets fed DON-contaminated diets consumed less feed, had lower live weight gain and consequently lower body weights at the end of the experiment. As visceral organs grow faster than the rest of the body, especially after weaning, the apparently increased relative stomach weights might simply reflect an allometric effect. 

Neither grinding fineness nor DON contamination of the diet influenced the mild gross macroscopical lesions (Score 1). These results are in contrast to other findings where finely ground feedstuffs caused pronounced gross-macroscopical stomach lesions including ulcera (e.g., [13,16,17,18,19,20,21]). The failure to induce overt ulcers in the present experiment might be due to several variables known or discussed as triggering factors, such as duration of the study, age of the pigs, diet composition and feeding technique, exposure to pathogens and other stressors [7]. The incidence of gastric ulcers might also include a genetic component as suggested by Flatlandsmo and Slagsvold [22] who observed differences between sire groups used in their experiments. 

While gross macroscopical findings revealed no differences amongst the groups of the present experiment the histological evaluation showed that both *pars nonglandularis* and *pars glandularis* were characterized by a more pronounced lymphoplasmacytic infiltration in groups FUS-fine and CON-fine when compared to group FUS-coarse.

Regarding *pars glandularis*, adequate regulation of the mucus layer is an important part of the first-line defense mechanisms against pathogens and antigens potentially harmful for the epithelium and the organism. Therefore, any imbalances in mucus turnover could trigger a local immune response resulting in recruitment and proliferation of immune cells. Feeding of coarse meal compared to pelleted feed was shown to enhance the proportion of acid mucins in the intestines which was discussed to have consequences for the adherence capability of bacteria [14]. Although this effect was not demonstrated for stomach mucus, it seems reasonable to assume that grinding fineness might have consequences for the mucus composition and thus protective properties in this part of the digestive tract. Moreover, different particle sizes were suggested to result in different epitope patterns which could contribute to local immune reactions [23]. 

At least the more pronounced lymphoplasmacytic infiltration of the gastric mucosa along with a higher abundance of lymphocyte follicles in groups fed the finely ground feed suggest that the local immune system had to tackle with invaded antigens. 

The detritus filled *foveolae* and tubes observed in these groups could be interpreted as necrotic lesions of epithelial cells giving rise to a facilitated entry of antigens and the triggering of the local immune response.

An increased incidence of lymphoplasmacytic infiltration of the *pars nonglandularis* without gross-macroscopical alterations was also observed by Madson, et al. [24] after feeding a diet with a DON concentration of 5 mg/kg whereby grinding fineness was not addressed in this study. DON is known to modulate immune responses in pigs [1,25], whereat a stimulation or a depression might evolve, depending on dose and duration of exposure [26]. In the present experiment, we examined the responsiveness of PBMC to a mitogenic stimulus *ex vivo* and found these circulating immune cells to react with a higher proliferation when DON-contaminated wheat was fed. Therefore, the question remains whether this DON-associated immune-stimulating effect in circulating PBMC also occurred locally in the stomach mucosa and was responsible for the higher incidence of lymphocyte follicles observed in group FUS-fine. Additionally, the results of the PCA would support an association between local immune response and stimulation ability of circulating PBMC as indicated by clustering together with lymphoplasmacytic infiltration of *pars nonglandularis*, lymphoplasmacytic infiltration of *pars glandularis* and pH-value in stomach chyme. The involvement of the pH-value in stomach chyme is pathogenetically interesting as no significant treatment effects were observed for this parameter. 

While our findings did not reveal any effect of treatments on chyme pH-values others found a significantly higher pH-value in caecal content of pigs fed coarsely ground feed [27]. This effect was associated with a lower dry matter content whilst the starch content remained unaltered. In contrast, an increased starch inflow into the caecum has been deduced from higher starch contents in caecal chyme of piglets fed coarsely ground feed [8]. This was associated with a significant increase in lactobacilli and Gram-positive cocci and proposed to alter the fermentative profile in the hindgut in favor of propionic and butyric acid. Our results suggest that the effect of grinding fineness on the fermentative pattern in caecum is generally less pronounced and further influenced by *Fusarium* infection of the wheat. The increase in butyric acid in caecum of group CON-coarse compared to group CON-fine resulted both from a rise in its proportion and from an enhanced total VFA concentration. The decrease in group FUS-coarse compared to its finely ground counterpart, however, was solely due to a reduced butyric acid proportion. As similar relationships for butyric acid were observed in jejunal chyme it might be deduced that dietary treatments affected the microbial community in small and large intestine similarly. 

The markedly lower concentration of total VFA measured in caecal chyme of piglets fed the diets containing the DON-contaminated wheat occurred independently of grinding fineness. This strongly suggests that it might be simply a reflection of the lower substrate flow to the caecum due to feed intake reduction observed both in group FUS-fine and FUS-coarse at the same order of magnitude. Besides feed intake level and associated substrate flows to the caecum a possible involvement of physico-chemical alterations might have contributed to the lower total VFA-concentrations measured in caecal chyme of FUS-fed piglets. In particular, increased activities of non-starch-polysaccharide (NSP) hydrolyzing enzymes were detected as a result of *Fusarium* infection whereby the proportion of soluble NSP and extract viscosity was enhanced [9]. Moreover, not only cell wall cleaving enzyme activities were augmented but also amylase and protease activities were increased. These effects might have consequences for both the accessibility of endogenous enzymes to their substrates within the small intestine and the proportion of nutrients escaping digestion and absorption up to the terminal ileum and thus entering the large intestine. Clinical-chemical serum characteristics revealed clear treatment effects both on total protein and albumin concentration whilst indicators of hepatocyte integrity such as GLDH, GGT, ASAT and total bilirubin remained inconspicuous. The decreased serum protein concentration observed in FUS-fed piglets independently of grinding fineness might be caused by a compromised protein synthesis in the liver as the main site of synthesis of serum proteins. Other reasons for a reduced serum protein concentration might include a blood loss which can be observed due to gastric ulcers. Moreover, a decreased serum protein concentration might also be caused by a relative hyper-hydration induced by an excessive intake of water which, however, cannot be proved as water consumption was not recorded. Furthermore, as gross macroscopic inspection of gastric mucosa did not reveal any overt ulcers, a blood loss into the gastric lumen as a cause for the decreased protein concentration seems less probable. The DON-associated decrease in serum protein concentration which appeared to be independent of grinding fineness can only partly be explained by the lower albumin content as it further decreased in group FUS-coarse compared to group FUS-fine. This grinding fineness associated effect was also observed after feeding the CON-diets albeit at a higher level. Moreover, low background DON-exposure (CON-fine vs. CON-coarse) was associated with larger differences in serum albumin concentrations than a higher DON exposure (FUS-fine vs. FUS-coarse). Looking at the effects of grinding fineness in particular, the steeper negative slope of the regression of serum DON concentration on albumin concentration in piglets fed finely ground diets (CON-fine and FUS-fine) compared to their counterparts (CON-coarse and FUS-coarse) suggests that albumin concentration responded more sensitively to increasing DON exposure when DON containing diets were fed in a finely ground form. 

While the effects of DON on albumin synthesis were inconsistent and demonstrated either a decrease [28] or no effect [29], grinding fineness was hardly examined with regard to albumin and protein concentration which were shown to remain uninfluenced [27]. Similarly, feeding of fattening pigs with DON-contaminated diets up to a concentration of 1.12 mg DON/kg, either in a mash or pelleted form, did not influence serum protein concentration [30]. Despite these controversial literature reports it seems to be necessary to examine the possible underlying mechanisms of the clear treatment effects on albumin concentration that were observed in the present experiment in more detail. Thus, direct measurement of albumin synthesis using isotope techniques could clarify whether differences in serum albumin concentration are due to a variation in synthesis. Moreover, recording of water consumption together with indicators of water balance could provide information and help explain treatment effects on serum albumin concentration. 

In conclusion, DON contamination of feed exacerbates the adverse effects of finely ground feed on stomach mucosal integrity of rearing piglets with possible consequences for the local and systemic immune system. Consequently, finely ground feed and DON might influence the immune system in an interactive manner. From a preventive viewpoint, it is recommended to avoid a too fine particle size distribution and to minimize DON contamination of feed for rearing piglets. 

## 4. Materials and Methods

The piglet experiment was conducted according to the European Community regulations concerning the protection of experimental animals and the guidelines of the Regional Council of Braunschweig, Lower Saxony, Germany (File number 509.42502/09-01.03).

### 4.1. Experimental Design

The experiment was planned according to a complete two by two 2-factorial design and included two diets containing *Fusarium*-toxin contaminated wheat (FUS) at a proportion of 20% to target a DON concentration of 5 mg/kg while the other two diets contained uncontaminated control wheat instead (CON). The intended DON concentration of 5 mg/kg was more than five times higher than the guidance value for the critical dietary DON concentration of 0.9 mg/kg [11] in order to ensure adverse effects as a precondition for studying the interactions between dietary DON-contamination and grinding fineness. 

Components of the two diet types were ground on a hammer mill either with a sieve width of 5 mm (coarse) or 3 mm (fine) with the same rotational speed. The four resulting diets and the piglet groups fed therewith were designated as CON-fine, CON-coarse, FUS-fine and FUS-coarse. 

### 4.2. Piglet Experiment

Crossbred piglets (“Bundeshybridzuchtprogramm”, BHZP, Germany) weaned at an age of 21 days were used for the experiment. A total of 16 castrated piglets were assigned to each of the four treatment groups. Four piglets were kept in each of the 16 slatted floor pens in an air-conditioned sub-unit of the pig house. The floor pens had a width of 85 cm, a depth of 173 cm and they were equipped with two nipple drinkers and an automatic feeder for dry feed with a width of 70 cm at the front of the box. The stable was air-conditioned and temperature was adjusted to 32 °C on arrival of the piglets and thereafter continuously decreased to 20 °C. The light–dark cycle included 12 h light and 12 h dark. Piglets were clinically monitored during the experiment.

The experiment started after a period of 10 d in which piglets were fed a commercial diet for weaned piglets. The mean piglet weights of the CON-fine, CON-coarse, FUS-fine and FUS-coarse groups were 7.8 ± 1.5 kg, 7.7 ± 1.1 kg, 7.7 ± 1.2 kg, and 7.7 ± 1.1 kg, respectively, at the beginning of the experiment. The experiment lasted 5 weeks and covered the typical period for rearing piglets under practical conditions. Individual body weight and feed consumption per box were recorded weekly. Diets were provided in a dry meal form and offered for *ad libitum* consumption. 

In addition, after 3 weeks of feeding the experimental diets, blood was collected from superficial neck vessels from 6 piglets per group. Serum and heparinized plasma were used for clinical-chemical analyses and for the *ex vivo* viability and activity assays performed with PBMC. 

At the end of the experiment, these 6 piglets of each group sampled previously for blood were slaughtered by bleeding after electrical stunning. Stomach, duodenum, jejunum, ileum, caecum, colon and rectum were quickly localized *in situ*, incised in the fundus region of the stomach and in the approximate mid third of the intestinal segments and pH-values were measured by using a pH-meter equipped with an insertion electrode (WTW pH 530, BLB, Brunswick, Germany). Ingesta was collected from the corresponding sites of the jejunum and the caecum and either used fresh for ammonia-N determination or kept frozen at −20 °C for later analysis of volatile fatty acids. Finally, the weights of the emptied stomach, of liver, kidneys, spleen and heart were recorded. Stomach mucosa was grossly inspected macroscopically and graded according to a scoring system after Betscher [31]: score 0—intact epithelium, smooth and glistening white surface; score 1—mild changes, partially bile staining and hyperkeratosis; score 2—moderate degree of hyperkeratosis and bile staining over entire surface; score 3—high-degree hyperkeratosis; and score 4—severe lesions and scarring. Finally, *pars nonglandularis* and adjacent *pars glandularis* of the cardia glandular region of the stomach were excised and further processed for histology.

### 4.3. Analyses

#### 4.3.1. Diets

Particle size distribution of the diets was determined by dry sieve analyses according to DIN 66165-1:1987-04 [32] and DIN 66165-2:1987-04 [33] employing sieves according to DIN ISO 3310/1 [34].

DON in diet samples was analyzed by high performance liquid chromatography (HPLC) coupled to a diode array detector (DAD) after a cleanup with immuno-affinity columns (IAC) (DONprep™, R-Biopharm Rhone Ltd., Darmstadt, Germany) according to slightly modified method as recommended by R-Biopharm Rhone [35]. The detection limit was 0.03 mg/kg and the recovery rate amounted to approximately 89%. 

Nutrients in diets were analyzed according to the methods of the VDLUFA (Verband Deutscher Landwirtschaftlicher Untersuchungs- und Forschungsanstalten, Germany). In particular, the following methods were applied: No. 3.1 for dry matter (DM), No. 4.1.1 for Kjeldahl Nitrogen, No. 5.1.1 for ether extract, No. 6.1.1 for crude fiber, and No. 6.5.2 for aNDF_OM_ [36].

#### 4.3.2. Chyme

Ammonia-N in fresh chyme was determined by a modified Conway-method as described in detail by Voigt and Steeger [37], using a micro-diffusion vessel. Volatile fatty acids in chyme were determined according to Geissler, et al. [38] by using a gas chromatograph (Hewlett Packard 5580, Avondale, PA, USA) with a flame ionization detector.

#### 4.3.3. Blood

Plasma samples were analyzed for DON and de-epoxy-DON (de-DON) by using an HPLC method as described by Valenta, et al. [39] with modifications. In brief, samples were incubated overnight with β-glucuronidase (Type H2, Sigma-Aldrich Chemie GmbH, Taufkirchen, Germany) at a pH of 5.5 and a temperature of 37 °C. Thereafter, samples were extracted with ethyl acetate using disposable ChemElut^®^ columns for liquid/liquid extraction containing diatomaceous earth (Varian Deutschland GmbH, Darmstadt, Germany). Extracts were cleaned up using IAC (DONtest™ of VICAM, Klaus Ruttmann GmbH, Hamburg, Germany). HPLC was equipped with an UV-detector for detecting DON and de-DON. The detection limits for both substances were approximately 2 ng/mL with mean recoveries of 87%–98% and 88%–101% for DON and de-DON, respectively. 

Total bilirubin, total protein, albumin, aspartat-aminotransferase (ASAT), glutamat-dehydrogenase (GLDH), and γ-glutamyl transferase (GGT) were determined in serum samples by using an automatic clinical chemistry analyser (Eurolyser CCA180, Eurolab, Austria).

For *ex vivo* examination of basal metabolic activity and concanavalin A (ConA) stimulated metabolic activity PBMC were isolated from heparinized blood, diluted with phosphate buffered saline (PBS) at a ratio of 1:1, by density gradient centrifugation using Biocoll separation solution (Biochrome AG, Berlin, Germany). Metabolic activity was determined by the 3-[4,5-dimethylthiazol-2-yl]-2,5-diphenyl-tetrazolium bromide (MTT) as described elsewhere [40,41]. The results of the test are reported as optical density of the ConA-stimulated and non-stimulated cells, and as ratio between both measures, defined as stimulation index (SI).

#### 4.3.4. Histology

Tissue samples of *pars nonglandularis* and *pars glandularis* of the stomachs were fixed in a 10% formaldehyde solution buffered with CaCO_3_ for at least 24 h and embedded in paraffin. Tissues were then sectioned at a thickness of 5 µm and stained with hematoxylin and eosin (H & E) for histological examination. 

The tissue sections were visually graded according to the occurrence of lymphoplasmacytic infiltration (score 1, 2 and 3 for low, medium and high degree, respectively) blind to the treatments. Scores are reported as cumulative scores of two evaluated slides. 

For technical reasons, the samples of group CON-coarse were lost. Therefore, only groups CON-fine, FUS-coarse and FUS-fine were evaluated.

### 4.4. Calculations and Statistics

Performance parameters, clinical-chemical serum characteristics, results of the cell culture tests and organ weights were evaluated according to a complete two by two 2-factorial design of analysis of variance (ANOVA) with cereal type (CT) (uncontaminated control wheat, CON, *Fusarium*-toxin contaminated wheat, FUS) and structure, resulting from different sieve widths of the hammer mill used for feedstuff grinding, (Str) (fine, coarse) and the interactions between CT and Str as fixed factors. Chyme characteristics (pH, volatile fatty acids) were analyzed according to a complete two by two by two 3-factorial design ANOVA with the intestinal segment (Seg, jejunum, caecum) and the corresponding interactions as additional fixed factors. A “Repeated” statement was applied to account for possible dependencies of a specific parameter measured both in jejunum and caecum of the same animal. 

Results are reported as LSmeans and pooled standard error of means (PSEM). Tukey test was used for *post-hoc* testing of differences between LSmeans in case of significance of the fixed factors. 

De-DON concentrations in serum and histological lesion scores were not normally distributed and were evaluated by the non-parametric Mann–Whitney U test. Corresponding results were presented as medians and ranges (minimum–maximum).

Finally, to visualize the relationships between the large number of variables in a two-dimensional space, a principal component analysis (PCA) based on correlations was performed additionally. 

The relationships between serum DON and albumin concentration were further examined by linear regressions and slope comparisons.

All statistics were performed using STATISTICA 12.0 (StatSoft, Inc. 2014, Tulsa, OK, USA).

## Figures and Tables

**Table 1:** Composition of the experimental diets (g/kg as fed).

	Diet Type			
	CON-Fine	CON-Coarse	FUS-Fine	FUS-Coarse
Components:				
Wheat	300.0	300.0	100.0	100.0
*Fusarium* toxin contaminated wheat	-	-	200.0	200.0
Barley	400	400	400	400
Maize	100.0	100.0	100.0	100.0
Soya bean meal	150.0	150.0	150.0	150.0
Soybean oil	10.0	10.0	10.0	10.0
Vitamins and minerals ^1^	10.0	10.0	10.0	10.0
Calcium carbonate	10.0	10.0	10.0	10.0
Sodium chloride	0.5	0.5	0.5	0.5
l-lysine-HCl	7.0	7.0	7.0	7.0
l-tryptophan	1.0	1.0	1.0	1.0
dl-methionine	2.9	2.9	2.9	2.9
l-threonine	2.3	2.3	2.3	2.3
Formic acid ^2^	6.0	6.0	6.0	6.0
Phytase ^3^	0.3	0.3	0.3	0.3
Calculated composition:				
Crude protein	160.5	160.5	160.5	160.5
Metabolizable energy (MJ/kg)	13.25	13.25	13.25	13.25
Lysine	12.6	12.6	12.6	12.6
Methionine plus cystine	7.9	7.9	7.9	7.9
Threonine	7.4	7.4	7.4	7.4
Tryptophan	2.8	2.8	2.8	2.8
Calcium	5.7	5.7	5.7	5.7
Total phosphorus	4.8	4.8	4.8	4.8
Sodium	1.4	1.4	1.4	1.4
Deoxynivalenol (mg/kg)	0.0	0.0	5.0	5.0
Analyzed composition:				
Dry matter	886.6	890.8	888.2	889.3
Crude protein (Kjeldahl-N*6.25)	160.1	160.5	157.3	164.1
Crude fat (Ether extract)	33.6	33.6	34.8	36.0
Neutral detergent fibre (aNDF_OM_)	153.1	154.4	148.2	147.9
Crude fibre	37.5	38.7	39.7	39.7
Deoxynivalenol (mg/kg)	0.2	0.2	3.4	3.6
Particle size distribution:				
>710 µm	37.5	60.6	41.7	68.5
125–710 µm	48.8	32.2	47.0	25.1
<125 µm	13.6	7.2	11.4	6.4
d_50_ (µm)	560	940	610	1130

^1^ Provided per kg of diet: Ca, 1.0 g; P, 1.4 g; Na, 0.9 g; Mg, 0.1 g; Fe, 75 mg; Cu, 15 mg; Mn, 40 mg; Zn, 50 mg; I, 1.0 mg; Se, 0.2 mg; Co, 0.4 mg; vitamin A, 10,000 IU; vitamin D_3_, 1000 IU; vitamin E, 50 mg; vitamin B_1_, 1.0 mg; vitamin B_2_, 3.1 mg; vitamin B_6_, 2.5 mg; vitamin B_12_, 20.0 µg; vitamin K_3_, 2.0 mg; nicotinic acid, 12.5 mg; pantothenic acid, 7.5 mg; choline chloride, 125 mg; biotine, 50 µg; folic acid, 0.5; vitamin C, 50 mg; ^2^ ACIDOMIX^®^ Formic 65 G, Röthel GmbH, Gudensberg, Germany; ^3^ ZY PHYTASE 5.000 (LOHMANN ANIMAL HEALTH GmbH & Co. KG, Cuxhaven, Germany) declared phytase activity (EC 3.1.3.26): 5.000 FYT/g.

**Table 2:** Performance of weaned piglets fed a fine or coarse ground uncontaminated control diet (CON) or a *Fusarium* toxin contaminated diet (FUS) (*n* = 4).

Cereal Type (CT)	Structure (Str)	Feed Intake (kg/d)			Live Weight Gain (kg/d)			Feed to Gain Ratio (kg/kg)		
		d 1–21	d 22–35	d 1–35	d 1–21	d 22–35	d 1–35	d 1–21	d 22–35	d 1–35
CON	Fine	0.439 ^ab^	0.965 ^b^	0.649 ^b^	0.243	0.684 ^c^	0.420 ^c^	1.869	1.430	1.552 ^ab^
CON	Coarse	0.477 ^b^	0.818 ^ab^	0.614 ^b^	0.264	0.565 ^b^	0.384 ^bc^	1.802	1.451	1.597 ^b^
FUS	Fine	0.370 ^a^	0.686 ^a^	0.496 ^a^	0.243	0.485 ^ab^	0.340 ^ab^	1.534	1.428	1.459 ^a^
FUS	Coarse	0.360 ^a^	0.752 ^a^	0.517 ^a^	0.216	0.451 ^a^	0.310 ^a^	1.711	1.690	1.666 ^b^
*p*-values										
CT		**0.024**	**0.009**	**0.004**	0.368	**0.001**	**0.003**	0.150	0.357	0.783
Str		0.700	0.483	0.829	0.890	**0.062**	0.134	0.698	0.276	**0.015**
CT × Str		0.517	**0.081**	0.436	0.378	0.279	0.899	0.394	0.349	**0.092**
PSEM		0.036	0.056	0.035	0.026	0.037	0.020	0.138	0.124	0.044

Abbreviations: PSEM = pooled standard error of means; ^a–c^ Values with no common superscripts are significantly different within columns (*p* < 0.05).

**Table 3:** Clinical-chemical serum characteristics of weaned piglets fed a fine or coarse ground uncontaminated control diet (CON) or a *Fusarium* toxin contaminated diet (FUS) (*n* = 6).

Cereal Type (CT)	Structure (Str)	Total Bilirubin (µmol/L)	Protein (g/L)	Albumin (g/L)	ASAT (U/L)	GLDH (U/L)	GGT (U/L)	DON (ng/mL)	De-DON ^1^ (ng/mL)
CON	Fine	1.9	50.5 ^b^	31.5 ^c^	28.3	2.6	24.7	2.1 ^a^	0.0 ^a^ (0.0–0.0)
CON	Coarse	1.9	50.5 ^b^	26.5 ^ab^	25.0	2.2	21.7	1.2 ^a^	0.0 ^a^ (0.0–0.0)
FUS	Fine	2.1	47.6 ^a^	28.2 ^bc^	25.3	2.4	21.3	23.4 ^b^	2.3 ^b^ (2.0–2.5)
FUS	Coarse	1.7	47.0 ^a^	23.9 ^a^	29.7	3.1	24.5	27.0 ^b^	2.4 ^b^ (1.3–3.4)
*p*-values									
CT		0.888	**0.003**	**0.038**	0.736	0.376	0.936	**<0.001**	
Str		0.357	0.771	**0.002**	0.840	0.651	0.979	0.423	
CT × Str		0.510	0.771	0.814	0.132	0.174	0.328	0.176	
PSEM		0.2	1.0	1.3	2.4	0.4	3.1	1.7	

Abbreviations: GGT = γ-glutamyl transferase; ASAT = aspartate-aminotransferase; GLDH = glutamate-dehydrogenase; DON = deoxynivalenol; de-DON = de-epoxy-DON; PSEM = pooled standard error of means; ^a–c^ Values with no common superscripts are significantly different within columns (*p* < 0.05); ^1^ Mann-Whitney-U-test (*p* < 0.05).

**Table 4:** pH values in the chyme of consecutive gastro-intestinal segments of weaned piglets fed a fine or coarse ground uncontaminated control diet (CON) or a *Fusarium* toxin contaminated diet (FUS) (*n* = 6).

Cereal Type (CT)	Structure (Str)	Segment (Seg)						
		Stomach	Duodenum	Jejunum	Ileum	Caecum	Colon	Rectum
CON	fine	3.33	4.87	6.63	6.67	5.83	6.35	6.62
CON	coarse	3.30	5.73	6.37	6.53	5.83	6.33	6.50
FUS	fine	3.80	5.67	6.15	6.68	5.88	6.58	6.52
FUS	coarse	3.75	5.50	6.42	6.65	5.93	6.07	6.53
...	...	3.55 ^a^	5.44 ^b^	6.39 ^c^	6.63 ^c^	5.87 ^b^	6.33 ^c^	6.54 ^c^
*p*-values								
CT	0.421							
Str	0.930							
Seg	**<0.001**							
CT × Str	0.631							
CT × Seg	0.836							
Str × Seg	0.925							
CT × Str × Seg	0.723							
PSEM	0.07							

Abbreviations: PSEM = pooled standard error of means; ^a–c^ Values with no common superscripts are significantly different within lines (*p* < 0.05).

**Table 5:** Fermentation characteristics in ileum and caecum of weaned piglets fed a fine or coarse ground uncontaminated control diet (CON) or a *Fusarium* toxin contaminated diet (FUS) (*n* = 6).

Cereal Type (CT)		CON	CON	FUS	FUS	...			*p*-Values					PSEM
Structure (Str)		Fine	Coarse	Fine	Coarse	...	CT	Str	Seg	CT × Str	CT × Seg	Str × Seg	CT × Str × Seg	
Parameter	Segment (Seg)													
NH_3_	Jejunum	3.15	1.42	2.60	2.05	2.30	0.346	0.793	**0.022**	0.861	0.299	0.542	0.504	0.70
(mMol/L)	Caecum	3.30	4.78	7.07	6.37	5.38								
VFA	Jejunum	16.7	20.7	21.8	21.7	20.2	**0.002**	0.521	**<0.001**	0.215	**<0.001**	0.750	0.327	2.6
(mMol/L)	Caecum	139.0	155.0	109.7	103.3	126.8								
C2	Jejunum	78.2	75.3	74.0	79.1	76.6	0.329	0.587	**<0.001**	0.180	0.120	0.941	0.295	0.9
(%)	Caecum	57.1	56.8	59.9	62.0	59.0								
C3	Jejunum	13.3	10.9	12.9	10.7	12.0	0.552	0.288	**<0.001**	0.748	0.539	0.796	0.718	0.9
(%)	Caecum	30.2	27.5	27.1	26.6	27.9								
Iso-C4	Jejunum	0.00	0.30	0.13	0.08	0.13	0.719	0.719	**0.021**	0.104	0.795	0.130	0.369	0.03
(%)	Caecum	0.30	0.28	0.35	0.22	0.29								
C4	Jejunum	8.5	13.0	12.4	10.0	11.0	0.488	0.479	0.748	**0.012**	0.186	0.719	0.529	0.5
(%)	Caecum	10.2	13.0	10.7	8.8	10.7								
Iso-C5	Jejunum	0.00	0.28	0.17	0.13	0.15	0.873	0.489	**0.017**	0.089	0.957	0.360	0.786	0.04
(%)	Caecum	0.28	0.38	0.42	0.28	0.34								
C5	Jejunum	0.00	0.25	0.40	0.00	0.16	0.934	0.519	**<0.001**	0.727	0.728	0.393	0.393	0.12
(%)	Caecum	1.78	2.03	1.52	2.07	1.85								

Abbreviations: NH3 = ammonia; VFA = volatile fatty acids; C2 = acetic acid; C3 = propionic acid; C4 = butyric acid; C5 = valeric acid; PSEM = pooled standard error of means.

**Table 6:** Relative organ weights (g/kg live weight) of weaned piglets fed a fine or coarse ground uncontaminated control diet (CON) or a *Fusarium* toxin contaminated diet (FUS) (*n* = 6).

Cereal Type (CT)	Structure (Str)	Liver	Kidneys	Spleen	Stomach	Heart
CON	Fine	27.0	5.5	2.7	8.9 ^a^	4.8
CON	Coarse	28.1	4.9	2.8	9.9 ^ab^	4.5
FUS	Fine	24.9	4.7	2.7	9.3 ^a^	4.8
FUS	Coarse	27.1	4.9	2.2	10.8 ^b^	4.8
*p*-values						
CT		0.174	0.167	0.422	0.132	0.329
Str		0.153	0.594	0.542	**0.006**	0.491
CT × Str		0.640	0.135	0.422	0.534	0.329
PSEM		1.1	0.3	0.3	0.4	0.2

Abbreviations: PSEM = pooled standard error of means; ^a–b^ Values with no common superscripts are significantly different within columns (*p* < 0.05).

**Table 7:** Cumulative histopathological scores of *pars nonglandularis* and *pars glandularis* of stomach of weaned piglets fed a fine ground uncontaminated control diet (CON-fine) or a fine or coarse ground *Fusarium* toxin contaminated diet (FUS-fine and FUS-coarse, respectively) (*n* = 6, medians and ranges).

	CON-Fine	FUS-Fine	FUS-Coarse
*Pars nonglandularis*			
Lymphoplasmacytic infiltration ^1^	2.5 ^ab^ (1.0–4.0)	3.0 ^b^ (3.0–3.0)	2.5 ^a^ (2.0–3.0)
*Pars glandularis*			
Lymphoplasmacytic infiltration ^1^	3.5 ^a^ (2.0–5.0)	4.5 ^b^ (4.0–5.0)	4.0 ^a^ (2.0–4.0)

^1^ Score 1, 2 and 3 represent low, medium and high degree, respectively. Scores of two slides were cumulated; ^a,b^ Medians with no common superscripts are significantly different within lines (*p* < 0.05).
